# Supplementary material for: Laryngeal mask airway in neonatal stabilization and transport: a retrospective study
Source: Eur J Pediatr. 2023 Jul 4;182(9):4069–75. doi: 10.1007/s00431-023-05089-8 (PMC10570173; doi:10.1007/s00431-023-05089-8)
Supplement: Supplementary file 1 — Supplementary file1 (DOCX 44 KB) [file 431_2023_5089_MOESM1_ESM.docx]

**TITLE:** **Laryngeal mask airway** **in neonatal stabilization and transport: a retrospective study**

**Authors:** Francesco Cavallin 1, Laura Brombin 2, Marialuisa Turati 2, Chiara Sparaventi 2, Nicoletta Doglioni 2, Paolo Ernesto Villani 3, Daniele Trevisanuto 2

**Affiliations:**

1. Independent statistician, Solagna, Italy

2. Department of Women’s and Children’s Health, University Hospital of Padova, Padova, Italy

3. Department of Pediatrics, Poliambulanza Hospital, Fondazione Poliambulanza, Brescia, Italy

**Address correspondence to:**

Professor Daniele Trevisanuto, MD, Department of Women and Children Health, University Hospital of Padova, Via Giustiniani, 3, 35128, Padova, Italy, phone 0039 049 8213545, e-mail [daniele.trevisanuto@unipd.it](mailto:daniele.trevisanuto@unipd.it)

**Running title:** laryngeal mask airway in transferred infants

**Key words:** laryngeal mask airway, neonate, transport

**List of Abbreviations**

ILCOR International Liaison Committee on Resuscitation

IQR Interquartile range

LMA Laryngeal mask airway

NICU Neonatal intensive care unit

**What is known**

- A supraglottic airway device may be used as an alternative to face mask during neonatal resuscitation.
- Health caregivers has a fast learning-curve with the laryngeal mask, which is also less invasive than the endotracheal intubation.
- The laryngeal mask may be considered by health caregivers of low-level hospitals with limited exposure on airway management, but literature provides little information on this aspect.

**What is new**

- In a large series of transferred neonates, laryngeal mask use was rare but increasing over time, and showed some heterogeneity among referring centers.
- The laryngeal mask was safe and lifesaving in “cannot intubate, cannot oxygenate” situations.
- Future prospective, multicenter research may provide detailed insights on laryngeal mask use in neonates needing neonatal resuscitation and postnatal transport.

**ABSTRACT**

**Background:** Laryngeal mask airway (LMA) may be considered by health caregivers of level I-II hospitals for neonatal resuscitation and stabilization before and during interhospital care, but literature provides little information on this aspect. This study reviewed the use of LMA during stabilization and transport in a large series of neonates.

**Methods:** This is a retrospective study evaluating the use of LMA in infants who underwent emergency transport by the Eastern Veneto Neonatal Emergency Transport Service between January 2003 and December 2021. All data were obtained from transport registry, transport forms and hospital charts.

**Results:** 64/3,252 transferred neonates (2%) received positive pressure ventilation with an LMA, with increasing trend over time (p=0.001). Most of these neonates were transferred after birth (97%), due to a respiratory or neurologic disease (95%). LMA was used before the transport (n=60), during the transport (n=1) or both (n=3). No device-related adverse effects were recorded. Sixty-one neonates (95%) survived and were discharged/transferred from the receiving center.

**Conclusions:** In a large series of transferred neonates, LMA use during stabilization and transport was rare but increasing over time, and showed some heterogeneity among referring centers. In our series, LMA was safe and lifesaving in “cannot intubate, cannot oxygenate” situations. Future prospective, multicenter research may provide detailed insights on LMA use in neonates needing postnatal transport.

**INTRODUCTION**

One of the priorities of the regional perinatal care programs is the centralization of high-risk deliveries in level III hospitals to prevent neonatal morbidity and mortality (1). Nonetheless, some infants born at level I-II hospitals require urgent transport to a tertiary neonatal care facility due to unpredictable problems after birth or because maternal transfer was not possible (2,3).

Respiratory diseases are the most frequent reason for postnatal transfers (4). Before the arrival of the transport team, the local health caregivers are responsible for the stabilization of the patients, but their skills and experience on some neonatal resuscitation procedures (such as intubation) may be suboptimal because of their low exposure to such emergency conditions (5).

The laryngeal mask airway (LMA) is a supraglottic device for the airway management during anesthesia or resuscitation maneuvers in both adult and pediatric patients (6). In neonates, LMA is safe (7) and reduces the need for intubation and the ventilation time (8). In addition, health caregivers has a fast learning-curve with the laryngeal mask, which is also less invasive than the endotracheal intubation (9-12). These advantages suggest that LMA may be considered by health caregivers of level I-II hospitals for neonatal resuscitation in interhospital care, but literature provides little information on this aspect (13, 14). This study reviewed the use of LMA during stabilization and transport in a large series of neonates who underwent postnatal transfer by an Italian regional service.

**MATERIALS AND METHODS**

***Study Design***

This retrospective study evaluated the use of LMA during stabilization and transport in infants who underwent emergency transport by the Eastern Veneto Neonatal Emergency Transport Service (EV-NETS) between January 2003 and December 2021. The study was enclosed in a project on neonatal transport which was approved by the Ethics Committee of the Azienda Ospedaliera di Padova (Protocol number 0021321). Parents gave their written informed consent for scientific use of clinical records.

***Patients***

We retrospectively evaluated all transferred neonates between January 2003 and December 2021 for inclusion in the study. The only inclusion criterium was receiving LMA during the stabilization immediately before the transport or during the transport. There were no exclusion criteria.

***Padova Neonatal Emergency Transport Service***

The transport service has been described in a previous publication (4). Briefly, it was established in 1999 and was fully operating since August 2000. It serves the Eastern Veneto Region with a population of over 2 million people and around 20,000 births/year in 20 maternal-neonatal wards. Around 180-200 emergency transports and 70 back-transfers are performed every year. The main transport vehicle is a ground ambulance, but special situations can be covered with a helicopter or a boat. The team includes a NICU neonatologist, a neonatal intensive care unit (NICU) nurse, a driver, and an assistant who are on-call for 24 hours. Since 2003, the LMA has been included in the equipment available to the transport team.

***Data Collection***

We collected information regarding the transferred patients (demographics and diagnosis), the transport process (referring center, receiving center, travel distance), the timing of the use of LMA (during the stabilization immediately before the transport or during the transport) and the outcome. All data were obtained from transport registry, transport forms and hospital charts. The transport chart of the Eastern Veneto Neonatal Emergency Transport Service has a dedicated space for recording any adverse events occurring during the whole process (before and during the transport). During data collection, such information was revised to identify device-related adverse effects (such as major bleeding or esophageal lesion) as relevant for the purpose of the study.

***Statistical analysis***

Data were summarized as median and interquartile range (IQR) (continuous data) or frequency and percentage (categorical data). The proportion of transferred neonates who received LMA ventilation was modelled over time using Beta regression and a p-value less than 0.05 was considered statistically significant. Statistical analysis was carried out using R 4.1 (R Foundation for Statistical Computing, Vienna, Austria) (15).

**RESULTS**

Overall, 64 out of 3,252 (2%) transferred neonates received positive pressure ventilation with an LMA in 2003-2021 (Figure 1). The proportion of transferred neonates who received positive pressure ventilation with an LMA increased over the time period (p=0.001) and ranged from 0.7% to 8.0% according to the referring centers.

Neonatal and transfer information is displayed in Table 1. Overall, most neonates were transferred after birth (97%) due to a respiratory or neurologic (asphyxia) disease (95%). Sixty neonates (93%) received ventilation with an LMA only before the transport. Three neonates (5%) presented a severe congenital upper airway malformation (Pierre Robin sequence) which did not allow intubation, hence were treated with LMA before and during the transport. One neonate (2%) with meconium aspiration syndrome was treated with LMA during the transport due to an accidental extubation in the ambulance. The percentage of infants with gestational age <34 weeks or birthweight <2 kg was 23% (15/64) in LMA-treated transferred patients and 36% (1,192/3,252) in all transferred neonates during the same period. Mechanical ventilation was included in the respiratory management of 25 neonates before the transport (39%) and 40 neonates during the transport (63%). No device-related adverse effects (such as major bleeding or esophageal lesion) were found during the review of transport registry, transport forms and hospital charts.

After a median length of hospital stay of 16 days (IQR 6-26), 27 neonates (42%) were discharged and 34 (53%) were back-transferred. Three neonates (5%) died at the receiving center: one very preterm infant (30 weeks gestation and BW 1,190 grams) was transferred for prematurity, asphyxia and renal failure, and died at 10 days of life; one extremely low birth weight infant (25 weeks gestation and BW 820 grams) was transferred for prematurity and respiratory distress syndrome, and died at 112 days of life; one late preterm infant (34 weeks gestation and BW 2,500 grams) was transferred for severe perinatal asphyxia, and died at 1 day of life.

Table 2 summarizes neonatal and transfer information for the subgroups of RDS patients (n=31), asphyxia patients (n=30), and those with gestational age <34 weeks or birthweight <2,000 grams (n=15).

**DISCUSSION**

The recent International Liaison Committee on Resuscitation (ILCOR) Consensus on Science and Treatment Recommendation suggested that a supraglottic airway device may be used as an alternative to face mask during neonatal resuscitation immediately after birth (16). According to a recent survey among the directors of 446 European neonatal units, the availability of LMA was reported in 56% of the delivery wards but only one director (0.2%) declared to use the LMA as primary interface for initial respiratory support (17). Our study evaluated the use of LMA in a large series of neonates who underwent postnatal transfer by an Italian regional service in 2003-2021. We found that LMA was used in few cases, mainly at birth, and with large heterogeneity among the referring centers. The difference in LMA use between our study and the survey may be partially explained by the inclusion of level III hospitals and the evaluation of LMA as primary interface in the survey. Of note, we believe that the large heterogeneity in LMA use among the referring centers may mirror the different experience of health caregivers on neonatal resuscitation procedures and the professional background (i.e. midwife, anesthesiologist or pediatrician) in level I-II hospitals. Despite such heterogeneity, LMA was safe as no device-adverse events were recorded in our series, in agreement with previous studies reporting a rare incidence of such events (7,18).

Although LMA was mainly used at the referring hospital, it was sometimes needed during the transport in case of “cannot intubate, cannot oxygenate” situations (neonates with severe congenital upper airway malformations) and in case of accidental extubation in the ambulance. Of note, the transport team attempted the intubation in all three cases of “cannot intubate, cannot oxygenate” suggesting that such procedure may fail even when performed by experienced neonatologists. We believe that such finding supports the inclusion of a neonatal LMA in the emergency bag of the neonatal transport team (13,19).

In addition, the increasing use of LMA over time in our study may be associated with the recommendations about LMA in international guidelines on neonatal resuscitation (20,21) and the growing evidence on the role of LMA (22,23). While we cannot estimate the proportion of neonates undergoing postnatal transfer who would benefit from LMA, we believe that it is reasonable to assume a larger use of LMA in the future.

In our series, some neonates with gestational age <34 weeks and/or birthweight <2,000 grams received effective positive pressure ventilation with LMA, although it is recommended in larger newborn infants (16). These data suggest that the neonatal size-1 LMA may be used in neonates with smaller gestational age, but the small sample size does not provide adequate support on such interpretation and the development of smaller LMA sizes remains a reasonable preference (16).

To our knowledge, this is the first study assessing the use of LMA in neonates who underwent postnatal transfer. The limitations of the study included the retrospective design (which restricted the availability of some data such as details on resuscitation interventions and times at birth, as well as experience of health care staff at referring centers), the lack of information about LMA being used as primary interface or after failure of previous attempts (using face-mask or intubation), and the limited sample size. Within such limitations, this study adds new data on LMA use in neonates born in level I-II hospitals and undergoing postnatal transfer, and provides useful information to health care professionals who are involved in neonatal transport.

**CONCLUSIONS**

In a large series of transferred neonates, LMA use during stabilization and transport was rare but increasing over time, and showed some heterogeneity among referring centers. In our series, LMA was safe and lifesaving in “cannot intubate, cannot oxygenate” situations. Future prospective, multicenter research may provide detailed insights on LMA use in neonates needing postnatal transport.

**Acknowledgements** We are very grateful to the Eastern Veneto Neonatal Emergency Transport Service (EV-NETS) team.

**Declarations**

**Funding:** None.

**Conflicts of interest/Competing interests:** The authors have no potential conflicts of interest to disclose.

**Availability of data and material:** All data generated or analyzed during this study are included in this article. Further inquiries can be directed to the corresponding author.

**Code availability:** not applicable.

**Authors' contributions:** FC contributed to study concept and data analysis, wrote the initial draft and critically reviewed the manuscript. LB contributed to study concept, data collection, writing of the manuscript and critically reviewed the manuscript. MLT contributed to study concept, data collection, writing of the manuscript and critically reviewed the manuscript. CS contributed to study concept, data collection, writing of the manuscript and critically reviewed the manuscript. ND contributed to study concept, data collection, writing of the manuscript and critically reviewed the manuscript. PEV contributed to study concept, data collection, writing of the manuscript and critically reviewed the manuscript. tDT conceived the study, contributed to data interpretation, wrote the initial draft and critically reviewed the manuscript. All authors approved the final manuscript as submitted and agree to be accountable for all aspects of the work.

**Ethics approval:** This study was approved by the Ethics Committee of the Azienda Ospedaliera di Padova (ref. Prot. n. 0021321/20.02.2020). Parents gave their written informed consent for scientific use of clinical records.

**Consent to participate:** Written informed consent was obtained from participants.

Not applicable as the study collected administrative data regarding equipment and procedures in the healthcare centers.

**Consent for publication:** Not applicable

**REFERENCES**

1. Paneth N, Kiely JL, WallensteinS MM, Pakter J, Susser M (1982) Newborn intensive care and neonatal mortality in low-birthweight infants. A population study. NEJM 307:149–155

2. Guidelines for Perinatal Care. American Academy of Pediatrics. Committee on Fetus and Newborn. Edited by American College of Obstetricians and Gynecologists. Committee on Obstetric Practice. 8th Edition, (2017).

3. Lupton BA, PendrayMR (2004) Regionalized neonatal emergency transport. Semin Neonatol 9:125–133.

4. Trevisanuto D, Cavallin F, Loddo C, Brombin L, Lolli E, Doglioni N, Baraldi E; Servizio Trasporto Emergenza Neonatale STEN Group (2021) Trends in neonatal emergency transport in the last two decades. Eur J Pediatr 180:635-641.

5. Guidelines for Perinatal Care. American Academy of Pediatrics. Committee on Fetus and Newborn. Edited by American College of Obstetricians and Gynecologists. Committee on Obstetric Practice. Seventh Edition, 2012.

6. American Society of Anesthesiologists Task Force on Management of the difficult airway (1993) Practice guidelines for management of the difficult airway. Anesthesiology 78:597-602

7. Pejovic NJ, Myrnerts Höök S, Byamugisha J, Alfvén T, Lubulwa C, Cavallin F, Nankunda J, Ersdal H, Blennow M, Trevisanuto D, Tylleskär T (2020) A Randomized Trial of Laryngeal Mask Airway in Neonatal Resuscitation. N Engl J Med 383:2138-2147.

8. Qureshi MJ, Kumar M (2018) Laryngeal mask airway versus bag-mask ventilation or endotracheal intubation for neonatal resuscitation. Cochrane Database Syst Rev 3(3):CD003314.

9. Gandini D, Brimacombe J (2004) Manikin training for neonatal resuscitation with the laryngeal mask airway. Paediatr Anaesth 14:493-494.

10. Carron M, Veronese S, Gomiero W, et al (2012) Hemodynamic and hormonal stress responses to endotracheal tube and ProSeal laryngeal mask airway for laparoscopic 10 Journal of International Medical Research gastric banding. Anesthesiology 117: 309–320

11. Pejovic NJ, Trevisanuto D, Lubulwa C, Myrnerts Höök S, Cavallin F, Byamugisha J, Nankunda J, Tylleskär T (2018) Neonatal resuscitation using a laryngeal mask airway: a randomised trial in Uganda. Arch Dis Child 103:255-260.

12. Mizumoto H, Motokura K, Kurosaki A, Hata D 82018) Introduction of laryngeal mask airway in Japan, and its rescue use for newborns. Pediatr Int 60 :954-956.

13. Trevisanuto D, Verghese C, Doglioni N, Ferrarese P, Zanardo V (2005) Laryngeal mask airway for the interhospital transport of neonates. Pediatrics 115:e109-11.

14. Stroud MH, Trautman MS, Meyer K, Moss MM, Schwartz HP, Bigham MT, Tsarouhas N, Douglas WP, Romito J, Hauft S, Meyer MT, Insoft R (2013) Pediatric and neonatal interfacility transport: results from a national consensus conference. Pediatrics. 132:359-66.

15. R Core Team (2022). R: A language and environment for statistical computing. R Foundation for Statistical Computing, Vienna, Austria. URL <https://www.R-project.org/>.

16. Yamada NK, McKinlay CJD, Quek BH, Rabi Y, Costa-Nobre DT, de Almeida MF, Davis PG, El-Naggar W, Fabres JG, Fawke J, Foglia EE, Guinsburg R, Hosono S, Isayama T, Kapadia VS, Kawakami MD, Kim HS, Lee H, Liley HG, Madar RJ, Nakwa FL, Perlman JM, Roehr CC, Rüdiger M, Schmölzer GM, Sugiura T, Trevisanuto D, Wyckoff MH, Wyllie JP, Weiner GM. Supraglottic airways for neonatal resuscitation (NLS#618/5340 [Internet] Brussels, Belgium. International Liaison Committee on Resuscitation (ILCOR) Neonatal Life Support Task Force. (available at <http://ilcor.org>).

17. Trevisanuto D, Gizzi C, Gagliardi L, Ghirardello S, Di Fabio S, Beke A, Buonocore G, Charitou A, Cucerea M, Degtyareva MV, Filipović-Grčić B, Georgieva Jekova N, Koç E, Saldanha J, Sanchez Luna M, Stoniene D, Varendi H, Vertecchi G, Mosca F, Moretti C; Union of European Neonatal and Perinatal Societies (UENPS) Study Committee (2022) Neonatal Resuscitation Practices in Europe: A Survey of the Union of European Neonatal and Perinatal Societies. Neonatology 119:184-192.

18. Trevisanuto D, Cavallin F, Nguyen LN, Nguyen TV, Tran LD, Tran CD, Doglioni N, Micaglio M, Moccia L (2015) Supreme Laryngeal Mask Airway versus Face Mask during Neonatal Resuscitation: A Randomized Controlled Trial. J Pediatr 167:286-91.e1.

19. Bansal SC, Caoci S, Dempsey E, Trevisanuto D, Roehr CC (2018) The Laryngeal Mask Airway and Its Use in Neonatal Resuscitation: A Critical Review of Where We Are in 2017/2018. Neonatology 113:152-161.

20. Wyckoff MH, Aziz K, Escobedo MB, Kapadia VS, Kattwinkel J, Perlman JM, Simon WM, Weiner GM, Zaichkin JG (2015) Part 13: Neonatal Resuscitation: 2015 American Heart Association Guidelines Update for Cardiopulmonary Resuscitation and Emergency Cardiovascular Care (Reprint). Pediatrics136 Suppl 2:S196-218.

21. Wyllie J, Bruinenberg J, Roehr CC, Rüdiger M, Trevisanuto D, Urlesberger B (2015) European Resuscitation Council Guidelines for Resuscitation 2015: Section 7. Resuscitation and support of transition of babies at birth. Resuscitation 95:249-263.

22. Qureshi MJ, Kumar M (2018) Laryngeal mask airway versus bag-mask ventilation or endotracheal intubation for neonatal resuscitation. Cochrane Database Syst Rev 3:CD003314.

23. Yamada NK, McKinlay CJ, Quek BH, Schmölzer GM, Wyckoff MH, Liley HG, Rabi Y, Weiner GM. Supraglottic Airways Compared With Face Masks for Neonatal Resuscitation: A Systematic Review. Pediatrics. 2022 Aug 11:e2022056568. doi: 10.1542/peds.2022-056568.

**FIGURE LEGENDS**

Figure 1. Proportion of transferred neonates who received positive pressure ventilation with LMA in 2003-2021 (the grey line shows the trend estimate)
